# Study on Effect of Germination on Flavonoid Content and Nutritional Value of Different Varieties of Chickpeas

**DOI:** 10.3390/foods14132157

**Published:** 2025-06-20

**Authors:** Jiyuan Xue, Jia Yang, Yongqi Yin

**Affiliations:** 1 College of Food Science and Engineering, Yangzhou University, Yangzhou 210095, China; mz120232144@stu.yzu.edu.cn; 2 Yangzhou Center for Food and Drug Control, Yangzhou 225000, China; jiajia82112001@163.com

**Keywords:** chickpeas, sprouts, flavonoids, germination, nutrition

## Abstract

Chickpeas (*Cicer arietinum* L.) were popular for their high nutritional profile and abundance of bioactive constituents, making them highly sought after in the consumer market. This investigation evaluated the impact of germination on the levels of total phenolics, total flavonoids, and other bioactive compounds, as well as free amino acids, soluble proteins, dietary fiber, and starch, in two chickpea sprout cultivars. The results demonstrated that germination significantly enhanced the concentrations of total flavonoids and phenolics in chickpeas. Compared to ungerminated seeds, the total flavonoid content in Xinying No. 1 and Xinying No. 2 sprouts increased by 3.95-fold and 3.25-fold, respectively, while total phenolic content increased by 2.47-fold and 2.38-fold. Germination also significantly augmented free amino acid, soluble protein, and total dietary fiber content while reducing resistant starch and insoluble dietary fiber. Concurrently, the bioaccessibility of essential nutrients was substantially improved, as indicated by enhanced solubility. This research provided valuable insights for optimizing the nutritional quality and bioactive compound content of chickpeas through sprouting technology. These results provided critical insights for optimizing the nutritional and functional properties of chickpeas via sprouting and established a scientific basis for the development of functional foods from germinated chickpeas, underscoring their potential to support dietary health and wellness.

## 1. Introduction

As one of the world’s important legume crops, the chickpea (*Cicer arietinum* L.) possessed a rich history of cultivation and demonstrated significant potential for worldwide consumption [1]. Renowned for their nutrient-dense profile, chickpeas were excellent sources of plant-based protein and dietary fiber while also harboring abundant bioactive compounds and antioxidants such as phenolic acids, flavonoids, and other polyphenolic substances. These phytochemicals conferred a range of health-promoting effects, such as anticancer and anti-inflammatory activities, as well as osteoporosis prevention [2,3,4]. As a result, the chickpea was widely recognized as a critical dietary staple and functional food ingredient in global nutrition.

Flavonoids, recognized for their nutritional and health-promoting properties, are prevalent throughout the plant kingdom, especially in legumes [5,6,7]. Flavonoids are phenol secondary metabolites produced by plants through phenylpropanoid metabolism. The appropriate intake of flavonoids is of great benefit to the human body, including in the alleviation of postmenopausal symptoms and inhibition of cancer cell proliferation [8,9]. Chickpeas, due to their high flavonoid content, were considered a valuable dietary source of these compounds.

However, due to the presence of anti-nutritional factors (such as phytic acid, tannins, and protease inhibitors) in traditional plant-based diets, the nutritional and functional components of chickpeas were not fully utilized historically. These anti-nutrients limited human absorption and resulted in the low digestibility of chickpea-derived nutrients [10]. With the increasing consumer demand for functional foods, recent research has focused on processing strategies to enhance the flavonoid content and bioavailability in chickpeas.

Considerable research has been devoted to optimizing processing methods to improve the bioavailability of plant nutrients. Germination, a straightforward and cost-effective bioprocessing technique, has been shown to markedly modify the physiological and biochemical properties of legumes by activating endogenous enzymatic systems, thereby impacting antioxidant activity, nutrient composition, and digestibility [11,12,13]. During the germination process, seeds regulate the anabolic metabolism of secondary substances such as flavonoids and, at the same time, promote the decomposition of large molecular substances (such as starch and protein) and generate small molecular peptides, free amino acids, etc., thereby enhancing the nutritional value [14,15,16]. Additionally, germination has been demonstrated to increase the phenolic compound content, improve the functional attributes of pulses for diverse food applications, reduce cooking times, and mitigate anti-nutritional factors [17,18,19]. Consequently, germination technology was regarded as a pivotal approach to enhance the comprehensive quality of legume-based foods. For instance, Saleh et al. [20] reported that germination significantly increased flavonoid and phenolic contents in soybeans compared to ungerminated controls. This treatment also enhanced protease and lipase activities, improved protein digestibility, and elevated soybean antioxidant capacity. Gunathunga et al. [21] found that germination boosted total polyphenol and flavonoid levels in mung beans and soybeans, enhanced antioxidant properties, and facilitated starch hydrolysis into digestible fractions. Yılmaz Tuncel et al. [22] observed that the germination of chickpeas significantly reduced the contents of anti-nutrients (such as phytic acid and trypsin inhibitors), increased the contents of phenolic compounds and antioxidants, and improved in vitro protein digestibility. Germination could usually be divided into short-term germination and long-term germination. The germination time of 24–72 h was considered short-term germination and the germination time of 72–168 h was considered long-term germination. Long-term germination might have a risk of microbial safety and the yield of raw materials would decrease [17,23]. Therefore, short-term germination was more commonly used in the production and processing process.

Based on detailed statistics released by China’s Ministry of Agriculture and Rural Affairs, the scale of chickpea cultivation in China has exhibited a sustained and steady growth trajectory over recent years, with planting areas predominantly concentrated in the western regions of the country. Among these regions, Xinjiang has firmly established itself as the nation’s largest chickpea producer, contributing more than 80% of the total national output. This growth in cultivation scale and yield reflects the increasing agricultural importance of chickpeas in China’s crop portfolio.

However, despite the upward trend in production, the processing technologies and product development pathways for chickpeas remain notably limited in diversity. Current industrial practices largely rely on traditional processing methods, which restricts the creation of value-added products and limits the expansion of chickpeas into new market segments. Concurrently, while a body of research has explored the impacts of germination on enhancing the nutritional profiles and bioconversion efficiency of legumes, systematic investigations specifically targeting newly developed chickpea varieties cultivated in China are still scarce. This gap in research has resulted in a lack of scientific guidance for optimizing germination protocols tailored to local varieties, thereby hindering progress in unlocking their full potential for food industry applications.

Collectively, these challenges, including processing bottlenecks and insufficient research on variety-specific germination techniques, have constrained the ability to optimize the utilization of Chinese native chickpeas within the food sector. Addressing these limitations will require collaborative efforts to diversify processing methodologies and to conduct in-depth studies on germination-induced transformations in newly cultivated varieties, ultimately paving the way for innovative food products and enhancing the economic viability of China’s chickpea industry.

This study took two newly cultivated chickpea varieties, Xinying No. 1 and Xinying 2, as the research objects and systematically explored the physiological and biochemical characteristics, flavonoid biosynthesis, antioxidant ability, free amino acid content, soluble protein content, dietary fiber content, and starch content. The results provide a technical reference for developing functional chickpea products.

## 2. Materials and Methods

### 2.1. Experimental Design

Chickpea seeds (Xinying No. 1 and Xinying No. 2) were obtained from the Xinjiang Academy of Agricultural Sciences in 2024. Prior to germination, the seeds were stored in a cool place at room temperature. An appropriate number of chickpeas were taken, washed with distilled water, and then soaked in 1% (*v*/*v*) sodium hypochlorite solution for 20 min. The disinfected chickpea seeds were rinsed with deionized water and then soaked at 25 °C for 12 h. The chickpea seeds were evenly distributed on a tray, sprayed with 10 mL of water every 24 h, covered with gauze, and placed in a 25 °C constant temperature germination box for 3 d. Samples were randomly collected after 12 h of soaking and after 3 d of germination to assess various parameters of chickpea seeds, soaked chickpeas, and sprouted chickpeas.

### 2.2. Determination of Contents of Total Flavonoids and Total Phenols

The total flavonoid content was measured according to Huang et al. [24]. The sample was homogenized with 80% ethanol, then subjected to ultrasonication and centrifugation. The supernatant was collected and diluted and the absorbance at 260 nm was recorded using a UV spectrophotometer (DR6000, Shanghai Ruishi Technology Co., Shanghai, China). The total flavonoid content was calculated by lignin production standard curve. The determination of the total phenol content followed the method described by Mencin et al. [25]. The chickpea sprouts were triturated with methanol and then the supernatant was extracted by centrifugation, mixed with Folin reagent and sodium carbonate, and incubated in the dark. Absorbance was measured at 765 nm, calculated according to the standard curve of gallic acid, and expressed as μg GAE/g FW.

### 2.3. Determination of Antioxidant Capacity

Chickpea sprouts were thoroughly homogenized in 80% methanol and then centrifuged at 8000× *g* for 10 min. The obtained supernatant was used for subsequent analysis. The scavenging capacities of 2,2-diphenyl-1-picrylhydrazyl (DPPH) and 2,2′-azino-bis(3-ethylbenzothiazoline-6-sulfonic acid) (ABTS) were measured following the protocol reported by Xue et al. [26]. In brief, 0.1 mL of the supernatant was mixed with 2.9 mL of DPPH solution, incubated in the dark for 30 min, and the absorbance at 517 nm was measured using a UV spectrophotometer. The DPPH radical scavenging activity was expressed as a percentage. For the ferric ion antioxidant power (FRAP) assay, the working solution was prepared by mixing 300 mmol/L acetate buffer (pH = 3.6), 10 mmol/L TPTZ, and 20 mmol/L FeCl_3_ in a 10:1:1 ratio according to Rumpf et al. [27]. Specifically, 3 mL of the FRAP reagent was mixed with 100 μL of the sample, incubated at 37 °C for 10 min, and the absorbance was measured at 593 nm. The ferric reducing capacity of the sample was quantified using a FeSO_4_ standard curve ranging from 0.1 to 1.0 mmol/L.

### 2.4. Determination of Free Amino Acid Content and Soluble Protein Content

The quantification of free amino acids was performed following the protocol described by Kowalska et al. [28]. The chickpea sprout samples were homogenized and extracted with trichloroacetic acid solution. The resulting supernatant was collected via centrifugation and subsequently filtered prior to analysis using an amino acid analyzer. Soluble protein content was assessed according to the method of Vershinina et al. [29]. A total of 0.5 g of samples were homogenized with 5 mL of water and centrifuged at 8000× *g* for 10 min and 0.1 mL of supernatant was mixed with 0.9 mL of water and 5 mL of Bradford solution. After 2 min incubation, the absorbance was measured at 595 nm. Standard curves were prepared with bovine serum albumin.

### 2.5. Determination of Resistant Starch Content

The contents of rapidly digestible starch (RDS), slowly digestible starch (SDS), total digestible starch (TDS), and resistant starch (RS) were quantified following the protocol described by Chen et al. [30]. Samples and distilled water were combined in a 50 mL centrifuge tube, sealed, and subjected to boiling for 25 min with intermittent vortexing. After cooling to 37 °C, 10 mL of sodium acetate buffer (0.5 M, pH 4.5) was added to the gelatinized sample. Porcine trypsin (4.5 g) was dissolved in 30 mL of distilled water and centrifuged at 3000× *g* for 15 min; the resulting supernatant was combined with 3.9 mL of starch glucosidase. A 5 mL aliquot of the mixed enzyme solution was introduced into the sample tube, which was then incubated with continuous shaking at 37 °C. Glucose release was quantified using the GOPOD assay kit and the concentrations of RDS, SDS, and RS were subsequently calculated. TDS was determined as the sum of RDS and SDS.

### 2.6. Determination of Dietary Fiber Content

A 1 g of the sample was accurately weighed and mixed with 50 mL of distilled water. The pH was adjusted to 6.0, and 100 μL of high-temperature α-amylase solution was added, and the sample was boiled in a water bath for 20 min. After the mixture was cooled to room temperature, the pH was adjusted to 7.0 and 100 μL of 10,000 U/mL of protease and 100 μL of 3000 U/mL of starch glucosidase were added. The enzymatic reaction proceeded in a 60 °C water bath for 20 min. The enzymatic lysate was centrifuged and filtered. The filter residue was dried and weighed to obtain the insoluble dietary fiber (IDF) content. The filtrate was precipitated with 4 times the volume of anhydrous ethanol. After centrifugation, the soluble dietary fiber (SDF) content was quantified. Total dietary fiber (TDF) is the sum of IDS and SDF.

### 2.7. Statistical Analysis

All experimental data were conducted in triplicate, and results are presented as means ± standard deviations. Statistical analyses were performed using SPSS 22.0 software. Comparisons between Xinying No. 1 and Xinying No. 2 were assessed using an unpaired student *t*-test while differences among seed, soaked, and germinated groups were evaluated by one-way ANOVA followed by Tukey’s post hoc test. A *p*-value of less than 0.05 was considered indicative of statistical significance.

## 3. Results

### 3.1. Effect of Germination Treatment on the Morphology of Sprouts

**Figure 1:**
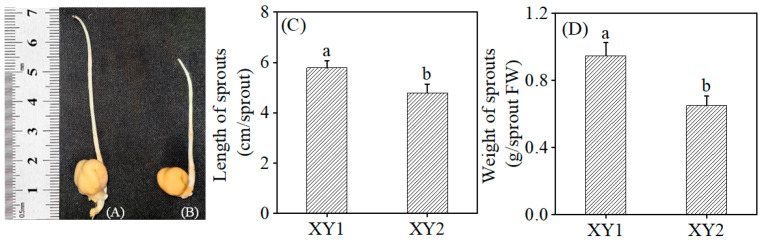
The effect of germination treatment on the morphology of Xinying No. 1 (**A**) and Xinying No. 2 (**B**). The effect of germination treatment on the length (**C**) and weight (**D**) of sprouts. Different lowercase letters represent significant differences between different varieties. XY1: Xinying No. 1; XY2: Xinying No. 2.

Figure 1 illustrates the morphology of the sprout, length of the sprout, and weight of the sprout of two cultivars (Xinying No. 1 and Xinying No. 2) following germination. It was evident that both the length and weight of Xinying No. 1 were significantly greater than those of Xinying No. 2.

### 3.2. Effect of Germination Treatment on the Content of Total Flavonoids and Total Phenolic Content

**Figure 2:**
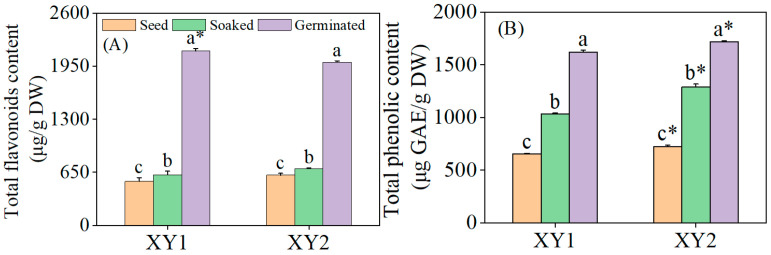
The effects of germination treatment on total flavonoid (**A**) and total phenolic (**B**) contents in chickpea sprouts. Different lowercase letters represent significant differences among different treatment of the same variety. Asterisks (*) indicate significant differences between different varieties under the same treatment conditions. XY1: Xinying No. 1; XY2: Xinying No. 2.

It can be seen from Figure 2 that the total flavonoid content in chickpeas experienced a significant increase after germination treatment (*p* < 0.05). Specifically, for Xinying No. 1, the total flavonoid content reached 3.95 times and 3.44 times compared to the content of seeds and after soaked treatment, respectively. In the case of Xinying No. 2, the total flavonoid contents of seeds and soaked treatment were 3.25 times and 2.88 times those before the corresponding treatments, respectively. Meanwhile, the total phenolic content also rose significantly after germination treatment (*p* < 0.05). Regarding Xinying No. 1, the total phenolic contents were 2.47 times and 1.57 times higher for seeds and after soaked treatment than before these treatments. For Xinying No. 2, the total phenolic contents of seeds and after soaked treatment were 2.38 times and 1.33 times those before the respective treatments. The results indicated that both germination and soaking treatment were capable of effectively enhancing the total flavonoid and total phenolic contents in chickpeas, with germination demonstrating a more prominent effect. After germination, the total flavonoid content of Xinying No. 1 was notably higher than that of Xinying No. 2. Conversely, the total phenolic content of Xinying No. 2 was significantly higher than that of Xinying No. 1.

### 3.3. Effects of Germination Treatment on the Content of Soluble Proteins

**Figure 3:**
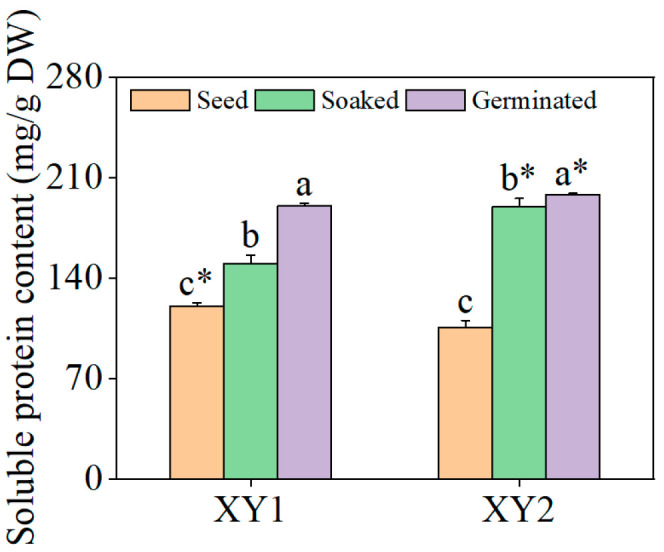
The effect of germination treatment on soluble protein content in chickpea sprouts. Different lowercase letters represent significant differences among different treatment conditions of the same variety. Asterisks (*) indicate significant differences between different varieties under the same treatment conditions. XY1: Xinying No. 1; XY2: Xinying No. 2.

It can be found from Figure 3 that it was clearly revealed that the content of soluble proteins in the two varieties underwent a significant increase following the soaking treatment (*p* < 0.05). Subsequent germination further augmented soluble protein levels, with values substantially surpassing those observed in the soaked samples (*p* < 0.05). When compared to the seed group, the content of soluble protein in Xinying No. 1 after germination treatment reached 1.58 times its original amount while, for Xinying No. 2, this figure was 1.87 times the original amount, indicating a pronounced enhancement in soluble protein accumulation in both varieties post germination.

### 3.4. Effects of Germination Treatment on the Content of Free Amino Acids

It can be seen from Table 1 that the total free amino acid content in Xinying No. 1 samples did not exhibit a statistically significant variation (*p* > 0.05). However, for Xinying No. 2, the total free amino acid content after the soaking treatment showed a significant increase compared to that of the seeds (*p* < 0.05), reaching 1.26 times the initial amount. Moreover, the total free amino acid content after the germination treatment was significantly higher than that of both the seed group and the soaked treatment group (*p* < 0.05), attaining 3.75 times and 2.98 times, respectively. In the case of Xinying No. 1, when compared with the seed and soaked groups, its total free amino acid content was significantly higher than that of Xinying No. 2 (*p* < 0.05). Nevertheless, after germination, the situation reversed, and the total free amino acid content in Xinying No. 2 was significantly higher than that of Xinying No. 1 (*p* < 0.05). After the germination process, the essential amino acid contents in both varieties witnessed a significant improvement when compared with their respective seeds and samples treated after soaking in water (*p* < 0.05). Specifically, the essential amino acid content in Xinying No. 1 was significantly higher than that in Xinying No. 2, reaching 1.92 times the original amount, which highlighted the impact of germination on the enhancement of essential amino acids in these two varieties. Specifically, regarding specific free amino acids, after germination, Lysine was the most abundant essential amino acid in both sprout varieties, with concentrations of 3457.08 μmol/g and 7686.47 μmol/g, respectively. Among all free amino acids, Arginine exhibited the highest content, reaching 17,435.18 μmol/g and 27,360.95 μmol/g, respectively.

### 3.5. Effects of Germination Treatment on Antioxidant Capacity

**Figure 4:**
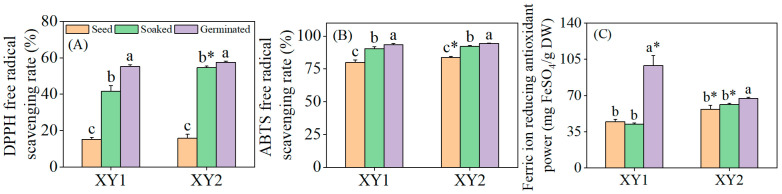
The effect of germination treatment on DPPH (**A**), ABTS (**B**), and FRAP (**C**) in chickpea sprouts. Different lowercase letters represent significant differences among different treatment conditions of the same variety. Asterisks (*) indicate significant differences between different varieties under the same treatment conditions. XY1: Xinying No. 1; XY2: Xinying No. 2.

It can be found from Figure 4 that after the soaking treatment, the DPPH radical scavenging rate and ABTS radical scavenging rate of the two varieties exhibited significant improvements when compared to the seed group (*p* < 0.05). However, the FRAP remained relatively unchanged, showing no significant difference (*p* > 0.05). Subsequent to germination, the antioxidant properties of both chickpea varieties were notably enhanced across all measured indices (*p* < 0.05). Notably, after germination, the FRAP of Xinying No. 1 was significantly higher than that of Xinying No. 2, which underscored the stronger overall antioxidant capacity of this particular variety. Collectively, these findings demonstrated that germination treatment can effectively augment the antioxidant properties of chickpeas, with observable variations in specific indices between the two varieties reflecting their distinct responses to the treatment.

### 3.6. Effects of Germination Treatment on the Content of Resistant Starch

**Figure 5:**
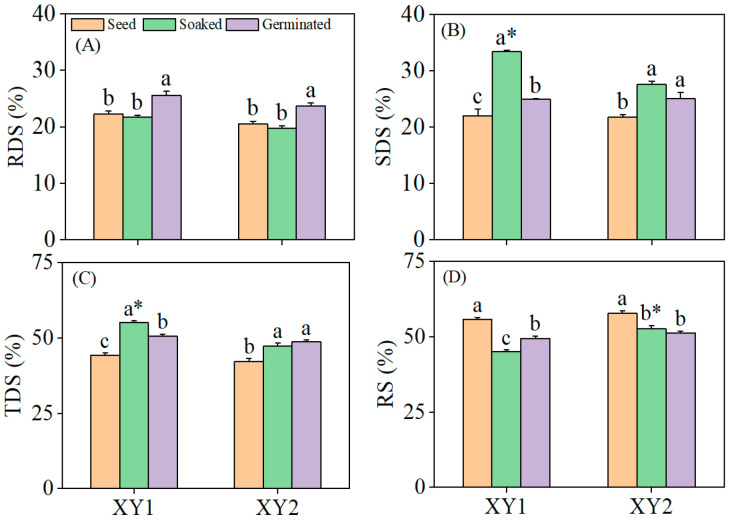
The effect of germination treatment on RDS (**A**), SDS (**B**), TDS (**C**), and RS (**D**) in chickpea sprouts. Different lowercase letters represent significant differences among different treatment conditions of the same variety. Asterisks (*) indicate significant differences between different varieties under the same treatment conditions. XY1: Xinying No. 1; XY2: Xinying No. 2.

It can be found from Figure 5, germination significantly elevated the RDS content in both chickpea cultivars compared to the seed and soaked treatment groups (*p* < 0.05). The SDS content reached its peak after the soaked treatment, demonstrating a significant elevation compared to the seed group (*p* < 0.05). Specifically, in Xinying No. 1, the SDS content in the soaked treatment group was notably higher than that in the germination group and also significantly exceeded the SDS content observed in Xinying No. 2. Both the soaked treatment and the germination treatment led to significant increases in the TDS content (*p* < 0.05), with Xinying No. 1 subjected to the soaked treatment exhibiting the highest TDS content among all groups. Conversely, the RS content in both chickpea varieties decreased significantly following both soaked and germination treatments (*p* < 0.05). These results collectively indicated that both the soaked treatment and the germination treatment were effective in enhancing the starch digestibility of the two chickpea varieties, with distinct treatment effects influencing the distribution of different starch fractions.

### 3.7. Effects of Germination Treatment on the Content of Dietary Fiber

**Figure 6:**
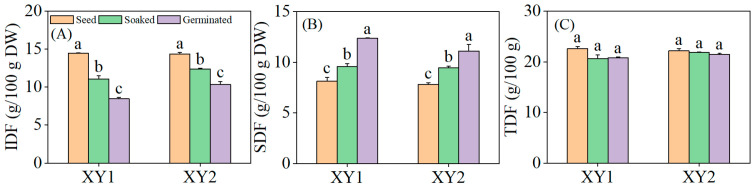
The effect of germination treatment on IDF (**A**), SDF (**B**), and TDF (**C**) in chickpea sprouts. Different lowercase letters represent significant differences among different treatment conditions of the same variety. XY1: Xinying No. 1; XY2: Xinying No. 2.

It can be found from Figure 6 that both soaking and germination treatments were found to significantly reduce the IDF content in chickpeas while significantly increasing the SDF content (*p* < 0.05). Notably, germination exhibited a more pronounced effect than soaking alone, demonstrating a superior capacity to modulate dietary fiber composition. Although there was a slight decrease in TDF content, this change did not reach statistical significance (*p* > 0.05). The results indicated that upon soaking or germination, the IDF in chickpeas underwent partial degradation and conversion into SDF, a process that enhances the digestibility and absorbability of dietary fiber by the human body. This modification ultimately improved the nutritional utilization efficiency of the chickpeas.

## 4. Discussion

Studies have shown that endogenous enzyme activity is activated during seed germination, promoting the decomposition of macromolecular substances and generating a large number of secondary metabolites [31]. In this investigation, the total phenolic contents of both chickpea varieties exhibited a marked increase following soaking treatment and were further augmented during germination. These findings indicated that soaking facilitates seed coat softening through water uptake and triggers pre-germination metabolic processes; however, its efficacy is constrained by the lag of energy metabolism and enzymatic activity. Both chickpea germination treatments significantly increased the total flavonoid content, by 3.95 and 3.25 times those of seeds, respectively, indicating that during the germination process, the production of ROS stimulated the styrene metabolism system and promoted the conversion of a large number of precursor substances to flavonoids [32]. In addition, in order to protect the growth and development of plants, the antioxidant system of the chickpeas was activated. In this study, soaked and germination treatment both improved the antioxidant ability of chickpeas. The difference was that soaking treatment improved the DPPH and ABTS capabilities but had no significant impact on the FRAP. The comprehensive enhancement of antioxidant properties after germination might have been due to the fact that with Rhizobia colonization during germination, the decomposition of cell walls enhanced the antioxidant ability of the chickpeas during germination [33]. In addition, during the germination process, the protease activity was enhanced, forming small molecule antioxidant peptides, further improving the antioxidant ability [34].

The activation of endogenous enzymes during germination not only regulated the metabolism of phenols and flavonoids but also significantly affected the hydrolysis of proteins and the dynamic balance of amino acids [35]. Research has shown that the seed endogenous enzyme system could be activated through processing, promote proteolysis, and increase the content of soluble proteins and free amino acids [36]. This study investigated the impact of soaking and germination treatments on soluble protein and free amino acid concentrations in two chickpea cultivars. The findings demonstrated that germination treatment markedly enhanced soluble protein levels, with Xinying No. 2 exhibiting a superior capacity for free amino acid accumulation post germination. Notably, the soluble protein content in both chickpea varieties following germination was significantly greater than that observed in the seed and soaked groups. It might have been so because the protease activity in the chickpeas was enhanced during germination, which prompted the storage proteolytic hydrolysis to small molecule peptides and soluble proteins, thereby increasing their contents [35]. The higher increase in Xinying No. 2 might have meant higher protease activity due to this variety’s properties. The total free amino acid content of Xinying No. 2 after germination was significantly higher than that of Xinying No. 1, and the amino acid content of Xinying No. 1 was higher in the seed and soaked stage. It might have been that the variety differences led to differences between amino acid synthase activities.

Starch consists of monosaccharides or glucose molecules, including amylose and amylose, and exists in plants in alternating crystallization layers in the form of particles [37]. Through processing technology, the content of phytic acid and anti-nutrition factors in plants could be reduced, the starch structure in plants could be improved, and the nutritional value could be increased [38]. Xing et al. [39] found that germination treatment could change the starch structure in quinoa. In addition, the TDS content increased under soaking and germination treatment, indicating that both soaking and germination treatment promoted the conversion of RS to RDS and SDS. This research studied the content of RDS, SDS, TDS, and RS in the two chickpeas under soaking and germination treatments. The results showed that the RDS content of the two chickpeas under germination treatments was significantly increased. This phenomenon may be attributed to the reduction in phytic acid and other inhibitory compounds in chickpeas following germination, which alleviates the suppression of amylase activity. The upregulation of endogenous amylase subsequently facilitates the hydrolysis of RS into smaller glucose molecules, thereby increasing the proportion of RDS. This observation aligns with the marked decrease in the RS content post germination. Conversely, the content of SDS was significantly higher after soaking compared to germination, potentially due to the degradation of SDS during the germination process.

Dietary fiber was an indigestive polysaccharide including non-starch polysaccharides, cellulose, pectin, hydrophilic colloids, oligomeric fructose, and resistant starch. It could also be divided into soluble and insoluble in a broad sense [40]. Processing technology could transform insoluble dietary fiber in plants into soluble dietary fiber that was easier to absorb [41]. Benítez et al. [42] found that germination can alter the dietary fiber structure in legume plants. In addition, during germination, the sprouts’ metabolic activity was enhanced and the IDF was consumed to support growth, which might have been the reason for the decrease in IDF content. This research measured the dietary fiber content in the two chickpeas under soaked and germination treatments and found that the TDF content in the two chickpeas did not change significantly, indicating that the conversion of IDF and SDF basically maintained a balanced total amount. The IDF content markedly declined while the SDF content increased following soaking treatment. This effect was likely attributable to the penetration of water into chickpea cell walls, leading to cell wall expansion and the disruption of the densely crystallized regions of the IDF. Nevertheless, as soaking does not activate the endogenous enzymatic system, the elevation in SDF content was less pronounced compared to germination treatment. During germination, endogenous enzymes such as cellulase, hemicellulase, and pectinase are activated in chickpeas, substantially facilitating the conversion of IDF to SDF.

## 5. Conclusions

This study confirmed that germination treatment effectively promoted the accumulation of nutrients of chickpeas, significantly improving the contents of biologically active compounds such as total flavonoids and total phenols in chickpeas. Following germination, the total flavonoid content in Xinying No. 1 and Xinying No. 2 reached 2138.0457 and 2000.2281 μg/g, respectively, while the total phenolic content reached 1620.0271 and 1720.5822 μg GAE/g, respectively. Both values were significantly higher than those observed in the ungerminated samples, thus enhancing the antioxidant properties of chickpeas. In terms of variety, Xinying No. 1 exhibited a significantly higher total flavonoid content compared to Xinying No. 2 whereas Xinying No. 2 had a markedly higher total phenolic content than Xinying No. 1. At the same time, germination treatment reduced the content of insoluble dietary fiber, resistant starch, etc. in chickpeas and improved the bioavailability of chickpea sprout seedlings. Therefore, it could be considered that chickpea sprouts are a good source of biologically active substances such as flavonoids and phenolic compounds and that the nutritional value and antioxidant activity of chickpeas after germination were significantly improved. Chickpea sprouts could be used as a good source of raw materials for nutritious foods.

## Figures and Tables

**Table 1:** The effect of germination treatment on the content of amino acids in chickpea sprouts. Different lowercase letters represent significant differences among different treatment of the same variety. Different upper letters represent significant differences between different varieties under the same treatment conditions. XY1: Xinying No. 1; XY2: Xinying No. 2.

Amino Acid Content (μmol/g DW)	XY1			XY2		
	Seed	Soaked	Germinated	Seed	Soaked	Germinated
Val	715.72 ± 7.63 ^Ab^	335.14 ± 101.78 ^Ab^	2354.43 ± 144.48 ^Ba^	247.26 ± 3.26 ^Bb^	239.65 ± 14.38 ^Ab^	5129.14 ± 56.00 ^Aa^
Met	60.63 ± 2.08 ^Ac^	71.07 ± 1.99 ^Bb^	308.72 ± 1.44 ^Ba^	56.7 ± 0.23 ^Ac^	118.26 ± 0.76 ^Ab^	683.61 ± 7.75 ^Aa^
lle	81.91 ± 3.07 ^Bb^	47.07 ± 8.04 ^Bc^	793.95 ± 16.47 ^Ba^	108.92 ± 0.73 ^Ab^	115.46 ± 6.05 ^Ab^	2062.53 ± 247.90 ^Aa^
Leu	73.02 ± 1.28 ^Ac^	181.21 ± 5.98 ^Bb^	583.14 ± 26.12 ^Ba^	76.75 ± 9.05 ^Ab^	222.89 ± 10.15 ^Ab^	1148.99 ± 271.93 ^Aa^
Phe	115.31 ± 3.61 ^Bc^	312.4 ± 9.59 ^Bb^	436.71 ± 45.98 ^Ba^	139.99 ± 1.31 ^Ab^	504.89 ± 9.58 ^Ab^	1059.97 ± 166.72 ^Aa^
Lys	1814.50 ± 29.46 ^Ab^	1516.45 ± 136.31 ^Ab^	3457.08 ± 49.93 ^Ba^	701.89 ± 3.98 ^Bb^	1250.89 ± 18.31 ^Bb^	7686.47 ± 554.26 ^Aa^
Total essential amino acid	2239.43 ± 302.24 ^Bb^	2529.02 ± 114.74 ^Ab^	19,645.08 ± 665.80 ^Aa^	3939.62 ± 212.41 ^Ab^	3085.31 ± 1046.05 ^Ab^	10,244.49 ± 1285.59 ^Ba^
Asp	1054.63 ± 13.82 ^Ab^	23.42 ± 2.93 ^Ac^	2402.4 ± 7.27 ^Aa^	792.48 ± 93.60 ^Bb^	11.27 ± 1.06 ^Bc^	1208.10 ± 143.55 ^Ba^
Ser	ND	457.92 ± 8.14 ^Ab^	4904.33 ± 150.16 ^Ba^	74.75 ± 6.45 ^Ab^	413.25 ± 48.09 ^Ab^	10,382.98 ± 650.28 ^Aa^
Glu	10,833.52 ± 624.17 ^Aa^	10,431.10 ± 338.78 ^Aa^	9807.53 ± 1505.84 ^Ba^	5629.04 ± 2.62 ^Bc^	7691.25 ± 50.08 ^Bb^	19,219.68 ± 659.12 ^Aa^
Gly	1065.52 ± 54.81 ^Aa^	982.33 ± 13.69 ^Aa^	559.01 ± 22.05 ^Bb^	671.85 ± 1.47 ^Bc^	994.84 ± 5.62 ^Ab^	1210.34 ± 47.32 ^Aa^
Tyr	105.64 ± 7.48 ^Ac^	263.34 ± 6.05 ^Bb^	352.48 ± 37.68 ^Aa^	91.79 ± 1.19 ^Ab^	318.03 ± 10.20 ^Ab^	460.39 ± 146.98 ^Aa^
Ala	983.88 ± 67.96 ^Ac^	1186.25 ± 7.25 ^Ab^	2994.6 ± 66.17 ^Ba^	736.93 ± 2.30 ^Bb^	738.25 ± 8.02 ^Bb^	5556.06 ± 670.87 ^Aa^
Cys	674.27 ± 19.19 ^Aa^	612.92 ± 46.18 ^Aa^	207.42 ± 9.38 ^Bb^	497.5 ± 12.23 ^Bb^	581.09 ± 3.70 ^Ba^	241.58 ± 0.01 ^Ac^
His	224.87 ± 7.69 ^Ab^	231.1 ± 6.26 ^Ab^	1776.47 ± 29.68 ^Ba^	140.33 ± 2.02 ^Bb^	231.48 ± 0.04 ^Ab^	3844.21 ± 316.55 ^Aa^
Arg	29,654.38 ± 756.66 ^Aa^	27,775.51 ± 1335.55 ^Aa^	17,435.18 ± 736.59 ^Bb^	14,154.8 ± 39.81 ^Bc^	16,419.55 ± 368.58 ^Bb^	27,360.95 ± 974.99 ^Aa^
Total amino acid content	45,686.01 ± 1741.07 ^Aa^	45,420.2 ± 167.93 ^Aa^	46,331.96 ± 2664.37 ^Ba^	24,058.23 ± 64.46 ^Bc^	30,244.27 ± 207.67 ^Bb^	90,248.94 ± 376.82 ^Aa^

## Data Availability

The original contributions presented in this study are included in the article. Further inquiries can be directed to the corresponding author.
